# A multicentre sero-behavioural survey for hepatitis B and C, HIV and HTLV among people who inject drugs in Germany using respondent driven sampling

**DOI:** 10.1186/1471-2458-14-845

**Published:** 2014-08-14

**Authors:** Ruth Zimmermann, Ulrich Marcus, Dirk Schäffer, Astrid Leicht, Benjamin Wenz, Stine Nielsen, Claudia Santos-Hövener, R Stefan Ross, Oumaima Stambouli, Boris-Alexander Ratsch, Norbert Bannert, Claus-Thomas Bock, Claudia Kücherer, Osamah Hamouda

**Affiliations:** Department for Infectious Disease Epidemiology, Division for HIV/AIDS, STI and Blood-borne Infections, Robert Koch Institute, Berlin, Germany; Department of Infectious Diseases, Division for HIV and other Retroviruses, Robert Koch Institute, Berlin, Germany; Department of Infectious Diseases, Division for Viral Gastroenteritis and Hepatitis Pathogens and Enteroviruses, Robert Koch Institute, Berlin, Germany; Deutsche AIDS-Hilfe, Berlin, Germany; Fixpunkt, Berlin, Germany; Institute of Virology, National Reference Centre for Hepatitis C, University Hospital Essen, University of Duisburg-Essen, Essen, Germany; Takeda Pharma Vertrieb GmbH & Co. KG, Jägerstrasse 27, 10117 Berlin, Germany

**Keywords:** PWID, Sero- and behavioural survey, hepatitis B, hepatitis C, HIV, HTLV, Respondent driven sampling, Second generation surveillance, Injecting drug users

## Abstract

**Background:**

People who inject drugs are at high risk for hepatitis B, hepatitis C and HIV. HTLV was reported by neighboring countries to be prevalent in this population, but the situation for Germany is unclear. To generate seroprevalence and related behavioural data and to enhance prevention efforts against these infections for drug users in Germany, a multicentre sero- and behavioural survey was initiated. People who inject drugs are not well reached by services for testing and counselling for blood-borne infections in Germany. An interventional part of the study is intended to prove feasibility and acceptance of testing and counselling in low-threshold drop-in settings.

**Methods/Design:**

Between May 2011 and March 2015, eligible participants (persons having injected drugs within the last 12 months, aged 16 years+, and living in the study city) are recruited by respondent driven sampling, using low-threshold drop-in facilities as study-sites in eight German cities with large drug scenes. Calculated sample size is 2,033 participants. Capillary blood samples collected as dried blood spots are anonymously tested for serological and molecular markers of hepatitis B and C, HIV, and HTLV I and II. A detailed face-to-face-interview about hepatitis- and HIV-related knowledge, former testing, imprisonment, sexual and injecting risk behaviour is conducted with participants. Staff is trained to offer pre- and post-test-counselling of blood-borne infections and HIV rapid testing to participants.

**Discussion:**

We chose respondent driven sampling for recruitment of participants to improve representativeness of results. Persons, who are not reached by the facility where the study is conducted, are aimed to be included by recruitment through their personal social network of injecting drug users. To reduce differential biases in the questions on knowledge of transmission and prevention of infections, we present true statements on hepatitis B, C and HIV, their possible routes of transmission and measures of prevention to participants. Participants are told that the statements are true and are asked to answer if they knew this fact already or if it is new to them. In case of knowledge gaps they are offered free targeted counselling as well as free HIV rapid testing and post-test counselling of HIV and hepatitis test results.

## Background

### Epidemiological situation of injecting drug users with regards to hepatitis B, C, HIV and HTLV in Germany

Like in other Western European countries, the epidemic of blood-borne and sexually transmitted infections in Germany is mainly driven by transmission in vulnerable groups. People who inject drugs (PWID) are one of the groups most affected by hepatitis C virus (HCV) infections, but also by hepatitis B virus (HBV) infections as well as human immunodeficiency virus (HIV), and often co-infections with several agents occur. Furthermore, PWID have been found to be at increased risk for infection with the human T-lymphotropic virus (HTLV).

#### Hepatitis B

HBV is highly contagious and is transmitted through parenteral, sexual and vertical routes. Globally, around 240 million people are chronically infected with hepatitis B, and about 600,000 people die every year due to the consequences of hepatitis B. Since 1982, a safe and effective vaccine exists to prevent hepatitis B infection [1]. In Western Europe, drug users are one of the key populations affected by HBV, with prevalence of chronic infections (HBsAg positive) ranging between 0.0% and 17.8% [2].

Germany is a country with low HBV prevalence. A recent population-based survey revealed an anti-HBc antibody prevalence of 5.1% in the general adult population due to former infection, and 0.3% (95% CI [0.2; 0.6]) show signs of current infection or carrier status [3]. Studies from former years indicate signs of former infection in 30–60% and low vaccination prevalence among PWID in German cities [4, 5]. Chronic HBV infection with the potential of virus transmission is estimated to be present in 1–7% of PWID [4, 5].

#### Hepatitis C

HCV affects approximately 170 million people worldwide and accounts for almost half a million deaths annually [6, 7]. Unlike most RNA viruses which usually cause acute diseases, HCV establishes persistent infections in more than 70% of affected persons leading to severe liver diseases like cirrhosis and hepatocellular carcinoma. Transmission of HCV occurs primarily via blood and blood-contaminated objects. It is known from experimental settings, that HCV can survive drying and environmental exposure at room temperature for at least 16 hours in infectious plasma [8]. Other studies confirmed the stability of HCV: environmental stability in suspension with viral infectivity was detectable for up to 28 days [9, 10]. However, in small volumes of < 2 μl which are volumes comparable with contaminated syringes, HCV remains detectable for at least 24 hours [11].

A recent population-based survey in Germany found a 0.3% prevalence of HCV infection in the general population [3]. Groups at increased risk like prisoners, hospitalised persons and PWID were not included in this survey, and migrants from regions with higher prevalence were underrepresented. Thus it must be assumed, that the true HCV prevalence is higher. Like in other Western European countries, in Germany HCV is hyperendemic in PWID, and regional studies found HCV prevalence of 50–80% in this group [2, 4, 12–17]. Notification data reveal that in recent years most infections with information on the mode of transmission occurred in PWID [18]. International studies identified several risk factors associated with HCV infection among PWID, such as years of injecting, sharing of needles, syringes and other equipment, HIV-co-infection and imprisonment [13, 15, 19–22]. To our knowledge, in Germany representative prevalence and associated behavioural data for HCV infection among PWID is missing, and it is not known to what extent co-infections occur in this group.

#### HIV

It is estimated that globally a total of 35,300,000 persons were living with HIV/AIDS in 2012 [23]. In Germany, like in other Western European countries, the HIV-epidemic is a concentrated epidemic where sub-populations, especially MSM are disproportionately affected [24]. The number of persons living with HIV/AIDS in Germany was recently estimated to be 78,000 by the end of 2012, of whom 65% are MSM [25]. PWID account for ~10% of infections among people who live with the virus [25]. New HIV infections among PWID have decreased in recent years, due to effective prevention measures like needle and syringe exchange programs (NSP), oral opioid substitution therapy (OST) and promotion of safer use. Among the estimated 3,400 new HIV infections in Germany in 2012, 210 (95% CI [160–270]) were transmitted by injection drug use, corresponding to 6.2% of new infections [25].

#### HTLV

HTLV is a retrovirus. Different genotypes have been described in humans, named 1–4. Worldwide 15 to 20 million persons are estimated to be infected with HTLV, mainly with the genotypes 1 and 2. Endemic regions are Japan, Western African countries, Latin America, Iran and the Caribbean [26]. In Europe, prevalence is much lower. Among first-time blood donors, a prevalence of 0–4.8 cases per 100.000 donors was reported for Scandinavian countries, 4.5–4.8/100.000 for France, the Netherlands and the United Kingdom, and 53/100.000 for Romania [27]. For many countries the epidemiological situation is unknown. It is expected that immigration from endemic regions may increase incidence and prevalence in European countries.

HTLV is the etiological agent of adult T-cell leukemia/lymphoma and HTLV-1 associated myelopathy/tropical spastic paraparesis. These diseases affect 3–5% of infected individuals. Specific antiviral treatment or vaccination is not available. HTLV is transmitted through similar transmission routes as HIV, mainly via blood, sexually and from mother-to-child by breastfeeding, and it is known that PWID are at high risk for infection with HTLV-1 and HTLV-2. Studies among PWID in the United States, in Spain, Ireland and Sweden revealed high HTLV prevalence up to 14% among PWID. [28–32] A recent sero-survey among drug users in Sweden found a HTLV antigen prevalence of 3.3% [32]. In Germany, HTLV-1 and -2 prevalence was estimated to be 0.7/10,000 pregnant women in 1997, but current and representative data for the general population or groups at increased risk are not available [33, 34]. The European Centre for Disease Prevention and Control (ECDC) recommends conducting HTLV sero-surveys if no representative data is available [35].

### Prevention of blood-borne infections among PWID in Germany

Interventions have been implemented in Germany, like in other countries, to reduce the risk of acquiring blood-borne viral infections among PWID, either by decreasing the frequency of injection by OST or by reducing the risk of infection by providing sterile needles and syringes via NSP. Both strategies have proven to be effective measures for reducing HIV transmission, and, if combined and provided at high coverage, as well for HCV transmission [36–39].

Another strategy implemented in recent years in some German cities is the prescription of injectable pharmaceutical diamorphine for subgroups of patients who are unable to stop injecting opiate drugs [40]. This measure not only serves to prevent overdose events and consequences of unpurified drug administration, but also facilitates social reintegration, because individuals do not need to “earn” their daily doses of drugs by risky or criminal acts, and results in the reduction of unsafe injections.

To monitor the impact of these programmes, it is essential to analyse in detail behavioural items combined with biological surveillance, ideally over time. By identifying gaps in knowledge of transmission and prevention of infections, as well as behaviours that favour transmission, targeted interventions may be derived from this study.

### Second generation surveillance

The WHO/UNAIDS guidance on second-generation surveillance for HIV infection strongly recommends the use of behavioural surveillance in the planning and evaluation of behavioural interventions, particularly in countries with low-level or concentrated epidemics [41].

Behavioural and socio-demographic information is essential in monitoring of blood-borne infections, as transmission of these infections in PWID strongly depends on specific risk behaviour, such as sharing of injection equipment and unprotected sex. These behavioural factors are influenced by socio-economic, policy and other factors, such as stigmatization, criminalization, marginalization, and limited access to prevention, treatment and care. Accordingly, also socioeconomic and structural information is important to survey in this group. Behavioural surveillance focused on PWID is recommended to investigate risk factors related to several viral infections with similar route of transmission [42]. Ideally, this is combined with biological data, to determine associations between risk or preventive behaviour and infection status.

The European Monitoring Centre for Drugs and Drug Addiction (EMCDDA) developed five key epidemiological indicators to achieve its goal of providing comparable information on drugs and drug addiction at European level. One of these is the drug-related infectious diseases key indicator (DRID), collecting data on the extent of infectious diseases — primarily HBV, HCV and HIV infection — among PWID. More recently, the collection of behavioural data as part of DRID was implemented [42–44].

### Respondent driven sampling

Respondent driven sampling (RDS) is a relatively new approach to recruit hard-to-reach populations for biological and behavioural surveillance. The precondition for using this recruitment strategy is the existence of social networks within the targeted population. Characteristics of hard-to-reach populations are the unknown size of the population and strong privacy concerns, because membership to these groups often involves stigmatized or illegal behaviour [45, 46]. Among PWID, social networks are driven by consumption of an illicit product and the need to maintain information about source, price and quality. Sharing equipment is also important for some individuals. Networks among PWID are therefore often well connected [46].

RDS is a modified chain-referral sampling technique, using statistical weighting for network sizes of individuals to generate representative estimates. Recruitment starts with eligible and pre-selected starter persons, representing different characteristics from the targeted population, the so-called “seeds”. These persons are trained to recruit up to three further persons willing to participate in the survey, and each participant will be asked to recruit further three eligible peers for participation.

The recruitment process is supported by a coupon system and primary and secondary incentives (for participation and for referral of further participants). Individual identification numbers allow for monitoring of the recruitment chains. In long chains with several “recruitment waves”, the composition of the sample begins to reach a point of “equilibrium”. The assumption is that the final sample will be independent of the characteristics of the first wave of seeds from whom recruitment began [47].

By asking participants detailed questions about network size and composition, statistical weights for each individual are generated. Persons with large network sizes have a higher probability of selection into the study and are therefore down-weighted while participants with smaller networks receive a greater weight for analysis [47].

### Multiplier method for population size estimation

Estimating the population size of a hidden and hard-to-reach population is challenging. Behaviours practiced by injecting drug users or opioid users are illegal, and thus, this group or other discriminated and stigmatized populations prefer not being identified or counted. Data on prevalence of injecting drug use in Germany are scarce, and estimations of population size in past studies used a multiplier method based on treatment data, police contacts and reported drug related deaths [48]. There are different approaches on how to estimate the prevalence of hidden and hard-to-reach populations while using the RDS methodology which are either based on two or more independent data sources. Methodologies are described in detail elsewhere [49, 50]. Two types of multipliers are commonly used with RDS, the “service multiplier” and the “unique object multiplier”. To obtain estimations on city-level, we want to use both methods, if possible, in the respective study cities.

### Objectives of the study

Objectives of the survey are to determine seroprevalence of HBV (including prevalence of vaccination acquired antibodies), HCV, HIV and HTLV I and II among PWID in the respective study cities and to estimate a mean seroprevalence for these infections among PWID in Germany. Molecular testing aims at identifying different genotypes of HCV and HIV among PWID in Germany. Furthermore we want to identify behavioural determinants, risk factors and characteristics of PWID and to evaluate knowledge and knowledge gaps of PWID concerning these infections and their transmission to recommend improved prevention measures and interventions against these infections among PWID. If possible, we want to estimate the prevalence of injecting drug use in study cities. Furthermore, the interventional part of the study intends to prove feasibility and acceptance of testing and counselling on blood-borne infections in low-threshold settings.

This cross-sectional study is also intended to build up a network of sites and facilities for regular monitoring of HBV, HCV and HIV among PWID in the future.

## Methods/Design

### Design of the study

This is a multicentre sero- and behavioural survey among PWID using RDS. We chose “DRUCK-Study” as name for the survey, an acronym of “**Dr**ogen **u**nd **c**hronische Infektions**k**rankheiten” (“Drugs and chronic infectious diseases”). “Druck” is at the same time the slang word for “shooting a drug”. The study is funded by the German Ministry of Health and coordinated by the Robert Koch Institute (RKI), which is the national public health institute with the mandate for infectious disease surveillance and related research.

The Federal Commissioner for Data Protection and Freedom of Information approved the study protocol on 29/11/2012 (III-401/008#0035). Ethics approval was received as of 19/11/2012 from the ethics committee at the medical university of Charité, Berlin (EA4/036/11).

### Selection of study cities

**Figure 1:**
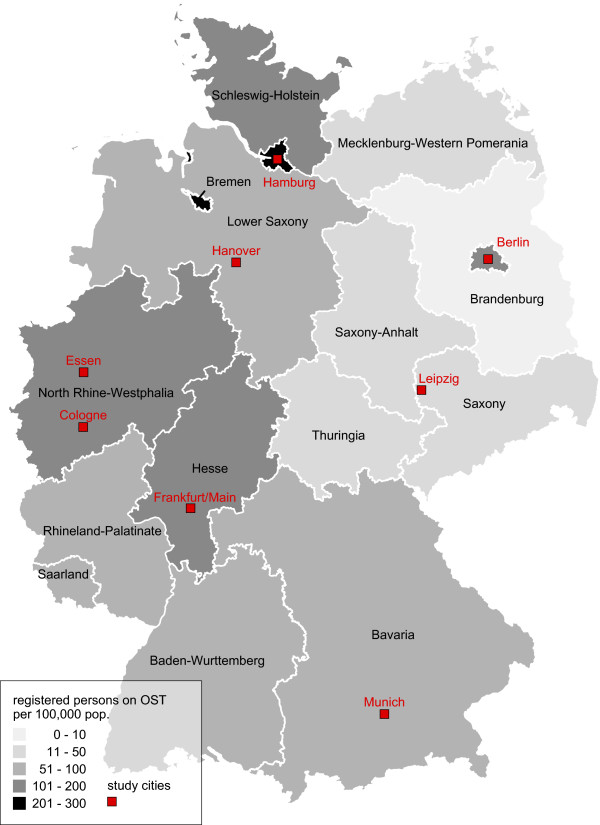
**Federal states of Germany by persons on OST per 100,000 population, and study cities.** Source: Bundesinstitut für Arzneimittel und Medizinprodukte/Bundesopiumstelle: Bericht zum Substitutionsregister 2014 http://www.bfarm.de/SharedDocs/Downloads/DE/Bundesopiumstelle/SubstitReg/Subst_Bericht.pdf?__blob=publicationFile&v=6 (last accessed July 4th, 2014).

The survey is conducted in several large German cities with a population of more than 500,000 inhabitants. The decision for a study site followed a list of criteria, that were collected during a situation analysis, such as the presence and size of a drug scene in the city, estimated prevalence of injecting drug use, number of persons on OST, existence of low-threshold facilities willing and able to participate in the study, interest of potential cooperating partners to set up structures for testing and counseling of infectious diseases in drug consumption rooms or other low-threshold drop-in facilities for injectors. Experts and representatives of these facilities from 10 large German cities were invited to a kick-off meeting in May 2012 to inform the coordinators at RKI about the respective local structures and to discuss the feasibility of conducting the DRUCK-Study. After this meeting, the following study sites were selected: Berlin, Essen (as pilot cities), Leipzig, Frankfurt, Cologne, Hanover, Munich, and Hamburg (Figure 1, Table 1).

**Table 1:** **Characteristics of selected study cities**

	Berlin	Essen	Leipzig	Frankfurt	Cologne	Hanover	Munich	Hamburg
**Pop. size 2012**	3.375.222	566.862	520.838	687.775	1.024.373	509.485	1.388.308	1.734.272
Estimated prevalence opioid users*	7,000 (2001)	3,000 (1998)	802" (2012)	6,000 (1998)	4,000 (1998)	3,000 (1993)	3,500 (2001)	12,000 (1993)
**Number persons on OST per 100,000 pop. (federal state) +**	138 Berlin	137 North Rhine- Westphalia	16 Saxony	113 Hesse	137 North Rhine- Westphalia	95 Lower Saxony	63 Bavaria	237 Hamburg
**Number drug related deaths (city level) (2012) ♦**	113	20	8	21	29	3	35	49

*source: Kraus et al., 2004 [51].

‘’source: Suchtbericht der Stadt Leipzig, 2013:

http://www.leipzig.de/news/news/suchtbericht-2013-liegt-vor-alkohol-bleibt-das-grte-problem/ (last accessed July 4th, 2014).

+source: Bundesinstitut für Arzneimittel und Medizinprodukte/Bundesopiumstelle: Bericht zum Substitutionsregister 2014 http://www.bfarm.de/SharedDocs/Downloads/DE/Bundesopiumstelle/SubstitReg/Subst_Bericht.pdf?__blob=publicationFile&v=6 (last accessed July 4th, 2014).

♦source: Bundeslagebild Rauschgift 2012: http://www.bka.de/nn_193360/DE/Publikationen/JahresberichteUndLagebilder/Rauschgiftkriminalitaet/rauschgiftkriminalitaet__node.html?__nnn=true.

### Participants and inclusion criteria

We aim to include persons older than 16 years who report having injected drugs in the previous 12 months and consuming their drugs in the respective city. For participation in the survey a person furthermore needs to be recruited by a PWID who already participated in the study and who gave him/her a coupon for participation. Coupons are coded with a unique identification number and are the only means of identification for study participants. No other personal data allowing identification of study participants are collected. All participants have to provide written informed consent.

### Recruitment method

The RDS recruitment chains in each city are started from 8 to 12 initial recruits (‘seeds’), eligible persons who are identified through employees of local low-threshold drug facilities. Seeds are selected to cover a broad range of PWID in the study city with respect to the following characteristics: gender, age, country of origin and preferred local drug facility in the city, reported HIV infection status, preferred consumed drug, experience of sex work and previous imprisonment. Furthermore, seeds should be persons who are well connected to other eligible persons and who are able to communicate the contents of the study. Seeds and all other participants receive three recruitment coupons for distribution to eligible peers in order to recruit further participants. All respondents receive EURO 10 as primary compensation for completing the survey and possible transportation fees as well as an additional secondary incentive (EURO 5) for each successfully recruited participant (up to three peers).

Depending on the estimated size of the drug scene, we determined an expected sample size in each city. Recruitment and enrollment is conducted until the expected sample size is reached or at a pre-determined date, after 8 to 10 weeks, depending on opening hours and feasibility at the study site. Low-threshold drop-in facilities in each study city serve as study sites, and the study operating hours are usually outside the regular opening hours of the facility to avoid solely recruiting regular clients of the facility.

### Indicators, questionnaire development and behavioural data

The study was implemented to capture a broad range of indicators in order to compare German data with other countries and PWID with other (vulnerable) populations. The EMCDDA provides a model questionnaire to respond to a list of core -, additional - and optional indicators in sero-behavioural surveys for drug-related infectious diseases among PWID. Furthermore, we aimed to be able to derive data for reporting of the Global AIDS response progress (GARP)/United Nations General Assembly Special Session on HIV/AIDS (UNGASS) and provide indicators used by ECDC. A list of indicators that are covered with this survey is shown in Table 2.

**Table 2:** **Indicators by ECDC, EMCDDA and GARP/UNGASS provided by the survey**

Contextual indicators	
Highest level of education	ECDC, EMCDDA
Country of birth/country of birth of parents	ECDC, EMCDDA
Sex/gender	ECDC, EMCDDA
Age	ECDC, EMCDDA
Current living status	ECDC, EMCDDA
Knowledge about HIV/AIDS and/or treatment	ECDC, GARP/UNGASS
Awareness of prevention activities	ECDC
**PWID specific indicators**	
**Sharing needles/syringes in last 4 weeks (includes both lending and borrowing as well as otherwise; only current IDUs)	ECDC, EMCDDA
**Sharing of any injecting paraphernalia in last 4 weeks excluding needles/syringes (includes both lending and borrowing as well as otherwise; only current IDUs)	ECDC, EMCDDA
**Recent HCV testing uptake (ever and in last 12 months)	ECDC, EMCDDA
Injecting frequency in last 4 weeks	ECDC, EMCDDA
Number of different sharing partners (in last 4 weeks; only sharing needles/syringes)	ECDC, EMCDDA
Number of sterile needles/syringes for personal use in last 4 weeks	ECDC, EMCDDA
Opioid maintenance treatment	ECDC, EMCDDA
Years since first injection	EMCDDA
Ever injected in prison	EMCDDA
Types of drugs consumed	ECDC, EMCDDA
Health status and health care access/usage	EMCDDA
**General indicators for STI surveillance common to all populations**	
Number of sexual partners in last 12 months	EMCDDA, ECDC, GARP/UNGASS
Condom use at last intercourse (in last 12 months)	EMCDDA, ECDC, GARP/UNGASS
Condom use at last intercourse (in last 12 months) with identification of type of partner: stable/casual/paid	ECDC, GARP/UNGASS
**Ever and recent HIV-testing uptake (in last 12 months)	EMCDDA, ECDC, GARP/UNGASS
Result of last HIV test (reported or measured)	ECDC
Having paid for sex in last 12 m	ECDC
Having been paid for sex/anal/vaginal sex to clients for money, drugs or other benefits in last 12 m	ECDC, EMCDDA
Use of condom at last paid intercourse (in last 12 months)	ECDC

**EMCDDA core indicator.

The initial questionnaire for the pilot study in Berlin was developed following the recommendations of EMCDDA, and – in co-operation with the Berlin-based low-threshold facility Fixpunkt e.V. and Deutsche AIDS-Hilfe e.V. - adapted for the German situation. It was pretested and adapted several times before and during the two pilot studies to ensure validity and reliability before it was used in the main study. The final questionnaire consists of 114 questions and 26 knowledge items. The questions are classified into 13 chapters on socio-demography, personal network, injecting history, consumption patterns, sharing of injection and drug preparation equipment, OST, sexual risks, imprisonment, testing history and reported HBV-, HCV- and HIV-status, health status, overdose history, sources of information and knowledge on infections and their transmission. The latter are presented to participants as true statements concerning transmission and prevention of HBV, HCV and HIV, and are introduced as such by the interviewer. The participant has to answer whether he/she ‘knew this already’, ‘didn’t know exactly’ or ‘didn’t know’. Interviewers are trained not to intervene or to give explanations during the interview, if participants have knowledge gaps. Instead the interviewer should mark the respective statement and offer targeted counseling in these areas by a trained test-counselor after the interview, even if test results are not requested by the person.

### Biological data collection and laboratory testing

Capillary blood samples are collected by a trained lab technician from each participant by finger prick. Blood drops are then spotted on filter cards with 10 partially pre-punched disks (Whatman 903, GE Healthcare Life Sciences, Freiburg, Germany) to prepare dried blood spots. From each person a minimum of six blood spots of ~ 30 µl is needed. Filters have to dry for at least three hours or overnight at room temperature. They are then sent to the laboratory in zip-locked plastic bags together with desiccants via regular mail. Every DBS filter card, already labeled with the unique identifier, is marked with a lab identifier, which is entered into the lab database. Upon receiving the filter cards at the laboratory, filter disks are fully broken out, transferred to reaction tubes and stored away at -20°C until testing. Antibodies are eluted from the DBS in phosphate buffered saline supplemented with 0.05% Tween 20, 3% new born calf serum at a dilution of maximally 1:15 with respect to the blood volume dried down.

DBS testing for this study has been validated during the pilot study in 2011 by the Institute of Virology, National Reference Centre for Hepatitis C, at University of Duisburg-Essen, Essen. During this period, all serological tests were performed on the ARCHITECT system (Abbott Diagnostics, Delkenheim, Germany). For molecular analyses, the artus HBV LC PCR (Qiagen, Hilden, Germany) and the VERSANT HCV RNA qualitative (TMA, Siemens Healthcare Diagnostics, Eschborn, Germany) assays were used, respectively. The salient results of this validation are described in detail elsewhere [52]. The Institute of Virology, University of Duisburg-Essen, performed all tests during the first two pilots in Berlin and Essen, in 2011. Since 2012, for the remaining six cities testing is performed in the Department for Infectious Diseases at RKI, for HIV and HTLV in the Division for HIV and other Retroviruses, for HBV and HCV in the Division for Viral Gastroenteritis and Hepatitis Pathogens and Enteroviruses. The protocol has since been moderately modified and was therefore re-validated. We validated commercially available EIA (Monolisa) and recombinant immunoblot (recomLine) test kits using various HBV and HCV positive serum samples and additionally 35 serum, plasma, and DBS samples of the study population drawn in parallel. For anti-HCV EIA and immunoblot the data showed that 2/35 (5.71%) samples differ in DBS (were negative) in contrast to the serum and plasma samples (were positive) while the immunoblot confirmed the serum/plasma results. Comparison of anti-HBc results showed 2/35 (5.71%) differences between DBS and serum/plasma samples. However, the analysis of anti-HBs antibody detection in DBS, serum and plasma revealed differences of 7/35 (20%) between DBS and serum/plasma. The seven anti-HBs negative DBS were all at the detection limit of the EIA for DBS showing very low anti-HBs antibody concentrations of <100 U/l.

#### Serological testing and molecular genome detection by (RT)-PCR techniques for HBV and HCV

For the detection of anti-HCV antibodies the Architect anti-HCV (during the pilot study) and the Monolisa Anti-HCV Plus Version 2 (Bio-Rad, Hercules, USA) assays are used. To confirm anti-HCV EIA-positive results respective samples are additionally tested using the recomLine HCV IgG strip-immunoassay (Mikrogen, Neuried, Germany). The anti-HCV test systems are performed strictly as recommended by the manufacturers.

Serological detection of anti-HBs and anti-HBc antibodies is performed using commercial available EIAs according to the manufacturer’s instruction. For (semi)-quantitative determination of anti-HBs antibodies the ARCHITECT anti-HBs (during the pilot study) and the Monolisa Anti-HBs Plus assays (Bio-Rad, Hercules, USA) are used. Qualitative anti-HBc detection was performed by ARCHITECT anti-HBc asay during the pilot study and since 2012 by the Monolisa Anti-HBc Plus assay (Bio-Rad, Hercules, USA). Isolated positive anti-HBs results without any additional positive serological and molecular HBV diagnosis argue for a successful previous HBV vaccination.

Nucleic acids are extracted from the DBS following the protocol as described [53, 54] or using the NucliSENS® miniMAG® platform and the corresponding Magnetic Extraction Reagents (BioMérieux, Boxtel, Netherlands) according to the manufacturer’s protocol. Extracted and purified viral nucleic acid is stored at -80°C until use. Sample processing (DNA/RNA extraction, template preparation, master-mix preparation) and PCR are done in separate laboratory rooms, which are all certified for molecular diagnostics using standard precautions to prevent assay contamination.

For detection of HBV DNA during the pilot study, the artus HBV LC PCR assay was used. Since 2012, detection of all HBV genotypes (A-H) is performed as described previously [52, 55] with minor modifications. In brief, extracted nucleic acids and the primers HBV-22, HBV-65, and HBV-66 were used to amplify a 408 bp region of the preS/S-region of the HBV genome. For the second round the primer pair HBV-24, HBV-41, and HBV-64 are used. PCR run is performed as described previously [55]. Lower detection limit for HBV genome detection is 100 IU/ml in DBS.

Detection of HCV genomes was performed using the VERSANT HCV RNA qualitative (TMA) assay (during the pilot study) and since 2012, a one-step RT-PCR technique (Qiagen, Hilden, Germany). The RT-PCR method amplified a 262 bp region of the 5′-NCR domain of the HCV genome. Pre-denaturation is performed at 50°C for 30 minutes and followed by 95°C for 15 minutes. The PCR amplification is for 40 cycles including: denaturing at 94°C for 30 seconds, annealing at 54°C for 30 seconds, extending at 72°C for 45 seconds and followed by a final extension for 10 min at 72°C. The nested PCR is initiated by a denaturation step at 95°C for 15 min and 35 cycles at 94°C for 30 seconds, 58°C for 30 seconds, 72°C for 45 seconds and followed by a final extension for 5 min at 72°C. Lower detection limit for HCV genome detection is 1 × 10^3^ IU/ml in DBS.

All test results are transferred into the study database and forwarded to the coordinators, provided with interpretation and diagnosis. Diagnoses of HCV and HBV results are made according to the German guidelines for the Prophylaxis, Diagnosis and Therapy of HCV [56] and HBV [51, 57].

#### Genotyping of HBV and HCV

Since 2012, for sequencing, the RT-PCR products are purified by Exo SAP-IT kit (USB Corporation, Ohio, USA) according to the manufacturer’s instruction. Sequencing reactions are performed using 1–5 μl purified PCR products and 0.5 μM of the primers HCV-240 and HCV-235 for HCV genotyping and the primers HBV-24 and HBV-41 for HBV genotyping. Sequencing results are analysed using BioEdit 9.7 software (http://www.mbio.ncsu.edu/BioEdit/bioedit.html) and Geneious Pro (Version 5.5.7, Biomatters Ltd, Auckland, New Zealand, http://www.geneious.com). The phylogenetic reconstruction of DNA sequences is carried out by MEGA 5 software [58]. For alignment and HBV- and HCV-genotyping, prototype HBV- and HCV-genotype sequences retrieved from the NCBI Gene bank are used.

#### Serological testing for HIV and HTLV and molecular testing for HIV

HIV-1/-2 antibodies are screened with the ARCHITECT HIV Ag/Ab combo assay (during the pilot study) and since 2012, with the 3rd generation Murex HIV 1.2.O ELISA (DiaSorin) and Genscreen HIV 1.2.O ELISA (Bio-Rad Laboratories GmbH) according to the manufacturer’s instructions. Samples reactive in both ELISAs are analysed by the immunoblot HIV Blot 2.2 (MP Diagnostics, DiaSorin). If HIV-2 antibodies are suspected (presence of indicator band) the HIV-2 specific NEW LAV Blot 2 (Bio-Rad) is performed. Evaluation of the immunoblot results is performed according to the manufacturer’s instructions.

HTLV-1/2 antibodies are screened using the Murex HTLV1 + 2 ELISA (DiaSorin). Reactive samples are confirmed by HTLV Blot 2.4 (MP Diagnostics, DiaSorin).

An external HIV and HTLV-1 positive control (Dried Plasma Spot) is used in each test run in addition to the internal test controls.

HIV molecular testing is performed for confirmed positive HIV-1 samples. HIV-1 RNA is extracted as described above. HIV-1 viral load is determined by an inhouse quantitative real time TaqMan LTR-RT-PCR (101 bp fragment of the HIV-1 LTR, HXB2 Acc. No. #K03455, coordinates 542–641) using 67 μl plasma equivalent input RNA. Filter dried serial dilutions of HTLVIIIB in HIV negative human plasma are used as concentration standard. The lower limit of quantification for HIV-1-RNA from DBS is 650 copies/ml. Samples with viral loads ≥6500 copies/ml are analysed using the ViroSeq HIV-1 genotyping system (Abbott, Wiesbaden, Germany) to amplify and sequence the viral protease and partial reverse transcriptase. Subtyping is performed using the REGA subtyping tool (http://dbpartners.stanford.edu:8080/RegaSubtyping/stanford-hiv/typingtool, version 3).

### Study flow and training of study staff

Before starting the survey in each city a two-day training workshop is held where staff is trained for the different core functions in the study: a coupon manager is trained to screen for inclusion criteria, to prevent multiple enrolment of PWID, to provide a unique identification number to each participant and to document the recruitment chains, to explain the recruitment process to participants, to provide them with numbered coupons for further recruitment and to pay out primary and secondary incentives. Interviewers learn to conduct the questionnaire-assisted interview via intensive face-to-face training and to offer counselling by a trained person in case of knowledge gaps. We trained the counsellors to provide voluntary counselling for viral hepatitis and HIV (rapid testing). A laboratory person is qualified to perform HIV rapid testing with the Vikia HIV 1/2 (Biomérieux) from capillary blood and to apply capillary blood samples on filter cards. A medical doctor is trained in post-test counselling. At each study site a study site manager is trained to overview the study flow, to manage adverse events and to inform participants of the contents of the study and to obtain their consent.

**Figure 2:**
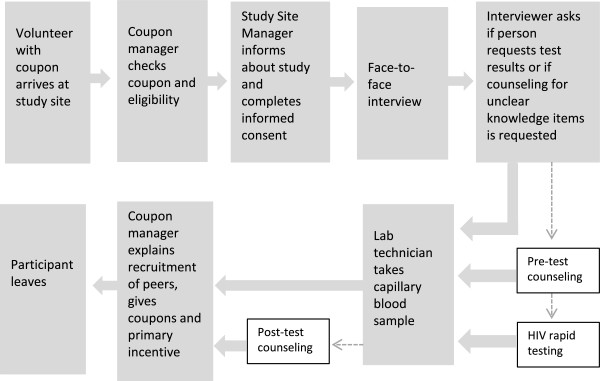
**Overview of the study flow.**

PWID coming with a valid coupon for study participation are first screened for inclusion criteria. Eligible participants are then informed about the objectives and contents of the survey: every participant has to undergo a detailed face-to-face interview and provide a capillary blood sample. Furthermore participants are offered voluntary counselling on HIV and viral hepatitis, anonymous HIV rapid testing, and they can request their HCV and HIV test results from DBS. After giving written informed consent, the participants receive the study documents marked by a unique numeric identifier and are then interviewed. The interviewer asks the participant if she/he wants to receive test results and whether anonymous HIV rapid testing is requested, and if yes, the person undergoes pre-test-counselling. In case of knowledge gaps during the interview the interviewer offers targeted counselling by trained staff to the participant. If the respondent does not want to receive test results or targeted counselling, she/he goes directly to the lab technician for finger prick. If requested, at that occasion the HIV rapid test is performed in supplement to the capillary blood sample. Study participation is completed after the capillary blood sample is taken. The participant hereafter returns to the coupon manager to receive the primary incentive. Furthermore, the coupon manager explains how to recruit up to three other persons for participation in the study and provides the participant with three numbered recruitment coupons.If the participant has had rapid testing for HIV with a reactive test result, a venous blood sample is taken for confirmatory testing in the laboratory. In that case, the participant has to come back after two weeks to receive the final result, as well as for HCV and HIV test results provided by DBS. Test results of rapid and DBS testing are generally given back to the participants by a medical doctor. Persons with newly diagnosed infections are referred to infectious disease specialists for confirmation of the test result by a venous blood sample and further medical care. An overview of study flow is shown in Figure 2.

### Sample size

We want to recruit current injectors in eight large German cities. Sample size was calculated on the basis of the infection with the lowest estimated prevalence (HIV). To our knowledge, estimates for HTLV I and II prevalence among German PWID are not available. Prevalence of HIV among PWID is expected to be 4%. The sample size should be large enough to ensure that if the true prevalence is 4%, the 95% CI of the resulting prevalence estimate should be included in the interval 2.5% to 5.5% with a probability of 90%. In case the true prevalence is 5%, we want the 95% confidence interval of the resulting prevalence estimate to be included in the interval 3.5% to 7.0% with a probability of 90%. To ensure this we need a sample size of 2,033 PWID.

We assume that the data of the different cities can be pooled for the estimation of a general HIV prevalence. Hence, it is sufficient to recruit 200 to 400 respondents per city, depending on the size of the respective drug scene.

### Data analysis

Data is double entered, using EpiData 3.1. RDS weights and 95% confidence intervals for sociodemographic factors and factors associated with infection are generated, using the RDS analysis tool (RDSAT Version 7.1) [53]. Frequency distributions will be tabulated for all variables, performing range checks and cross-validations. Characteristics of the study population will be described precisely, using RDSAT-generated weights for bivariate analysis, for the whole sample, but also for each study city separately. Multivariate logistic regression will be used to explore the associations between outcome variables such as RDSAT-generated weighted status of infection and vaccination, and risk- or protective factors such as opioid substitution treatment, drug consumption patterns, sharing of equipment, injecting in prison, unprotected sex, and socio-demographic factors (like educational level, living status, job situation). All analyses will be conducted using RDSAT Version 7.1 and the statistical software Stata, version 12.

## Discussion

### Choice of the RDS sampling strategy

Former studies among drug users in Germany were conducted as convenience sample in drug consumption facilities, or among persons in OST-facilities or detoxification units [4, 12–14, 54, 59]. We aimed to reach a more representative sample of the whole drug scene in a city and chose RDS, because this method claims to achieve representativeness. PWID are known to be a well-networked group, and RDS proved to be an effective method to rapidly recruit PWID [46, 50, 60, 61]. A long recruitment process over months would not be feasible in most study sites. Furthermore, RDS makes it possible to recruit PWID who might not be reached by the existing low-threshold facilities in the study cities and thus can only be reached by their personal social network. Collecting information about the PWID currently not reached is vital for improving local prevention efforts as well as for the planning of future regular monitoring of HBV, HCV and HIV among PWID by using data collected through these facilities.

### Asking questions on knowledge

Initially, in the pilot cities, the knowledge section of the questionnaire was designed questions on transmission and prevention of HBV, HCV and HIV with true and false response options that participants had to choose. Due to concerns about interviewer bias as well as for ethical considerations - realising that the knowledge questions presented an opportunity of learning for the participants - the design was changed. After discussion with experts during the kick-off-meeting of this study in May 2012, true statements concerning transmission, prevention, treatment and general characteristics of HBV, HCV and HIV are presented to participants. By providing true statements, and also introducing them as such, participants now have the opportunity to learn something while completing the interview. In case of knowledge gaps, participants are offered to see a counsellor, even if test results are not requested by the person. Learning facts about transmission and prevention of blood-borne viral diseases during the interview and being counselled in case of knowledge gaps is one of the interventional parts of the DRUCK-study. First experiences with this kind of knowledge statements prove feasible and well-accepted by both study staff and participants.

### Pre- and post-test counselling in low-threshold drug facilities

Until now, voluntary counselling and testing (VCT) for blood-borne viruses in Germany is mainly offered by medical doctors, substitution therapy specialists, and by some local health departments (anonymously and free of charge). Due to the illicit nature of drug consumption, PWID are not well reached by these services. A pilot project offering anonymous HIV rapid testing and counselling in some German drop-in-facilities showed that this group was reached successfully [62].

We conduct this survey in low-threshold drug facilities, where VCT for HBV, HCV and HIV was rarely available before. Before starting recruitment, staff is trained for all VCT procedures. Counselling follows international recommendations and the German recommendations provided by Deutsche AIDS-Hilfe [63]. If well-accepted during the study by both, staff and injectors, implementation of a regular VCT offer and of rapid testing in the facility may be eased.

### Challenges and limitations

There are a number of important challenges and limitations preexisting when designing the study. PWID are a stigmatized, hard-to reach population. Although all data is anonymised, and no personal information is collected from study participants, participants might have difficulties in reporting sensitive data such as unsafe use and sexual behaviours, imprisonment or testing history. We try to limit the reporting bias of sensitive data by choosing interviewers who are familiar to most of the local PWID (e.g. social workers or volunteers working at the local facilities). Behavioural surveys cannot take place without the informed consent of the respondent. However, signing a form might inhibit the feeling of anonymity. Thus, we offer participants to give oral consent to the study site manager, if written consent is refused.

Another issue concerns the question of true answers in self-reported behavioural data due to social desirability, and also of accuracy in items with 12 months recall periods.

Furthermore, potential selection bias might be a problem in our study population. Experience has shown that respondents are more likely to refuse to participate if they are asked to provide a specimen for testing [41]. Reaching the targeted sub-sample size for the respective study cities depends on many factors and might not be possible for each city. To reach the targeted total sample size (2,033), it might be necessary to conduct the study in an additional city.

In chronic infections like HBV, HCV and HIV infections it is not possible to draw direct associations between behavioural items and time of acquisition of infection. We can only identify associations between the status “infected” or “not infected” and risk and preventive behaviours reported for a certain time period in the past. In case an infection was newly diagnosed in the study we may assume that the same behaviour caused infection. If an infection was known already by the participant, direction of cause and effect could be both ways.

Because of the cross-sectional nature of this survey, we will be unable to infer direct causal relationships between socio-demographic and behavioural factors or the impact of prevention programs on behaviour change.

Moreover, a change of laboratory methodologies could limit direct comparison of test results of the two pilot cities and the remaining six cities. The dilution of the original sample volume during elution from the filter disk was 1:10 for the first two cities and then increased to maximally 1:15 for the following six cities to allow repeated testing of samples. With respect to HIV serology, established HIV infections are detected at comparable sensitivity by the two assays, however, individuals in a very early seroconversion stage could be missed by third generation EIA [50% of samples in seroconversion (reactive EIA/indeterminate immunoblot] resulted in false-negative immunoblot results, but still showing reactive/indeterminate ELISA]. The prevalence of HIV could therefore be slightly underestimated in 6/8 cities, but seroconverters are expected to represent only a very small fraction of the study participants.

We furthermore had to modify the anti-HBV and anti-HCV antibodies test systems after the pilot study. All methodologies were validated, but direct comparison between study sites has to be done with caution. Assessment of all serological test systems for HBV and HCV shows good accordance between directly tested serum samples in comparison to DBS except for anti-HBs in weakly positive sera. Weakly anti-HBs positive samples could be missed with this procedure. Thus the prevalence of anti-HBs antibodies, e.g. of HBV vaccinated individuals, based on the DBS technique, must be considered as minimal estimate. However, the anti-HCV and anti-HBc results demonstrate high accuracy of our test systems and yielded comparable validation results.

This is the first sero-behavioural survey among current injectors in Germany aiming to collect representative data on blood-borne viral infections in several cities in over 20 years. Knowledge from behavioural, serological and molecular testing data will help to focus prevention strategies. Depending on the outcomes, we will recommend conducting similar surveys in regular intervals, to monitor changes in behaviour and prevalence over time and assess outcomes of adapted preventive measures.

## Abbreviations

CI: Confidence interval
DRID: Drug related infectious diseases
DRUCK-study: Drugs and chronic infectious diseases study (Studie zu “Drogen und chronischen Infektionskrankheiten”)
ECDC: European Centre of Disease Prevention and Control
EIA: Enzyme immunoassay
EMCDDA: European Monitoring Centre for Drugs and Drug Addiction
GARP: Global AIDS response progress
HBV: Hepatitis B virus
HCV: Hepatitis C virus
HIV: Human immunodefiency virus
HTLV: Human T-lymphotropic virus
NSP: Needle and syringe exchange programme
OST: Opioid substitution treatment
PWID: People who inject drugs
RDS: Respondent driven sampling
UNAIDS: Joint United Nations Programme on HIV/AIDS
UNGASS: United Nation General Assemby Special Session
VCT: Voluntary counselling and testing
WHO: World Health Organisation.
